# Protocols for genomic epidemiology and source attribution of enteric bacteria causing diarrhoeal disease across Africa

**DOI:** 10.1136/bmjopen-2025-109566

**Published:** 2026-06-25

**Authors:** Henry Badji, Ben Pascoe, Madison P Goforth, Evangelos Mourkas, Evariste Bako, Isidore JO Bonkoungou, Modeste T Gampene, Marguerite Edith Malatala Nikiema, Barthélemy S Zoma, Ousman E Bah, Eustacia Jane Cassell, Abdoulie K Ceesay, Bubacarr E Ceesay, Ousman Ceesay, Bakary Conteh, Lamin Drammeh, Binta Faye, Abdoulie F Jallow, Fatima Jallow, Ousman Jallow, Samba Juma Jallow, Pa Modou Joof, Modou Kandeh, Mehrab Karim, Jarra Manneh, Abdou Ceesay, Ebrima Fofana, Mamud Jallow, Modou B Jarju, Samba Bah, Dodou Sanyang, Demba B Jallow, Mukaila I Alebiosu, Atanyiwoen Brusah, Akosua Bonsu Karikari, Courage KS Saba, Kaisa Haukka, C Matilda (Tilly) Collins, Adrian W Leach, Polina Levontin, Shani UP Ali, Frances M Colles, Matthew David Hitchings, Martin Antonio, Abdul Karim Sesay, Ozan Gundogdu, M Jahangir Hossain, Samuel K Sheppard

**Affiliations:** 1 Department of Infection Biology, London School of Hygiene & Tropical Medicine, London, UK; 2 Medical Research Council (UK) (MRC) Unit, The Gambia at the London School of Hygiene & Tropical Medicine, Banjul, Gambia; 3 Faculty of Veterinary Medicine, Chiang Mai University, Chiang Mai, Thailand; 4 Ineos Oxford Institute for Antimicrobial Research, University of Oxford, Oxford, UK; 5 Department of Biology, University of Oxford, Oxford, UK; 6 Zoonosis Science Center, Uppsala University, Uppsala, Sweden; 7 Laboratory of Molecular Biology, Epidemiology and Monitoring of Bacteria and Virus Transmitted by Food (LaBESTA), Universite Joseph Ki-Zerbo, Ouagadougou, Burkina Faso; 8 Department of Livestock Services, Government of the Gambia, Abuko, Gambia; 9 Ministry of Health, Regional Health Directorate, Government of the Gambia, Banjul, Gambia; 10 National Agricultural Research Institute, Banjul, Gambia; 11 Department of Clinical Microbiology, University for Development Studies, Tamale, Ghana; 12 Faculty of Biosciences, University for Development Studies, Tamale, Ghana; 13 Department of Microbiology, University of Helsinki, Helsinki, Finland; 14 Centre for Environmental Policy, Imperial College London, London, UK; 15 Department of Life Sciences, University of Bath, Bath, UK; 16 Medical Microbiology and Infectious Diseases, Swansea University Medical School, Swansea, UK

**Keywords:** BACTERIOLOGY, Gastrointestinal infections, Community child health, Protocols & guidelines

## Abstract

**Abstract:**

**Introduction:**

Enteric bacterial pathogens are a major cause of diarrhoeal disease in low-income and middle-income countries, with complex transmission pathways involving human, animal and environmental reservoirs. Conventional epidemiological and microbiological approaches provide important insights into pathogen burden and distribution but lack the resolution needed to characterise fine-scale diversity, antimicrobial resistance (AMR) and transmission dynamics. Whole-genome sequencing offers high-resolution tools to investigate these processes within a One Health framework.

**Methods and analysis:**

The Genomic Epidemiology and Transmission of *Campylobacter* in Africa (GETCampy-Africa) study uses a multicountry, One Health design to investigate pathogen diversity, source attribution and transmission pathways. The study uses a case–control framework, recruiting children with medically attended diarrhoea and asymptomatic community controls across sites in Burkina Faso, Ghana and The Gambia. Samples were collected from human participants, domestic animals and environmental sources following standardised protocols. Participant enrolment and sample collection have been completed, while laboratory processing, sequencing and genomic analyses are ongoing. Genomic data are analysed to assess population structure, AMR profiles and probabilistic attribution of isolates to potential reservoirs using comparative genomics and machine learning approaches.

**Ethics and dissemination:**

Ethical approval was obtained from relevant national and institutional committees in all participating countries. Written informed consent was obtained from participants or their guardians prior to enrolment. Findings will be disseminated through peer-reviewed publications, stakeholder engagement activities and open-access platforms to support public health interventions and policy development.

**STRENGTHS AND LIMITATIONS OF THIS STUDY**

- This study presents a harmonised, multicountry One Health genomic epidemiology framework for enteric pathogens in low-income and middle-income countries.
- Integration of clinical, environmental and animal sampling enables probabilistic source attribution across reservoirs.
- Use of whole-genome sequencing and machine learning provides high-resolution insights into pathogen diversity and transmission.
- Standardised protocols across sites enhance comparability but require adaptation to local resource constraints.
- The cross-sectional design limits inference on transmission directionality and temporal dynamics.

## Introduction

 Diarrhoeal diseases remain a major global health challenge, contributing significantly to illness and death among children under five, particularly in low-income and middle-income countries (LMICs).^1^,^6^ Africa bears a disproportionate burden of these diseases, driven by multiple interrelated factors, including limited healthcare infrastructure, poor sanitation and insufficient access to safe drinking water.^7^,^11^ The aetiology and epidemiology of diarrhoeal disease in LMICs contrast markedly with those in high-income countries (HICs), with different reservoirs, transmission dynamics and population structures. Perhaps the most important difference is that far less is known about diarrhoeal disease in these low-income settings.^12^,^16^

In LMICs, children are exposed early and repeatedly to enteric pathogens with high rates of asymptomatic carriage and environmental exposure.^6^ This can lead to a chronic cycle of reinfection with frequent subclinical and polymicrobial infections, and contrasts with diarrhoea in HICs which is usually acute, symptomatic and linked to foodborne outbreaks or travel.^17^ Common enteric bacterial pathogens include *Campylobacter*,^18^ *Shigella*,^19^ *Salmonella*^20^ and pathogenic *Escherichia coli*,^21^ all of which have been linked to prolonged diarrhoea in early childhood in LMICs.^22^,^27^ However, while transmission is well understood in HICs, the epidemiology in LMICs appears more complex, shaped by overlapping environmental, behavioural and socioeconomic risk factors. Recent evidence of antimicrobial-resistant (AMR) *Campylobacter* infection across five continents further highlights the global importance of understanding pathogen evolution, emerging strains and transmission dynamics.^28^,^34^

Landmark studies such as the Global Enteric Multicenter Study (GEMS)^35^,^37^ and the Malnutrition and Enteric Disease Study (MAL-ED)^38^,^40^ have substantially advanced understanding of diarrhoeal disease burdens, pathogen distribution by age and associations with growth outcomes.^41^,^48^ These studies, however, primarily relied on conventional culture or molecular diagnostics, which are effective for identifying pathogens, estimating burden and characterising diversity at higher taxonomic levels and provided lower-resolution genomic insights into pathogen diversity, transmission pathways and AMR.^49^ Whole-genome sequencing (WGS) provides complementary resolution at the within-species and strain level, and augments, rather than replaces, established epidemiological and microbiological methods. They also did not include systematic environmental or animal sampling, which is critical to a One Health understanding of pathogen reservoirs. Recognising these limitations, several recent studies have been applying genomic and metagenomic approaches to understand the complexity of enteric pathogen transmission across the human–animal–environment interface.^50 51^ These efforts align with the One Health paradigm and aim to bridge clinical and environmental microbiology, leveraging advances in sequencing technology and data science to generate actionable insights.

Several prior One Health enteric studies in Africa provide important context for Genomic Epidemiology and Transmission of *Campylobacter* (GETCampy). These include the *Campylobacter* Genomics and Environmental Enteric Dysfunction (CAGED) study in Ethiopia,^49 52^ the Pathogen Transmission and Health Outcome Models of Enteric Disease (PATHOME) in Kenya^52^ and the Urban Zoo in Kenya.^53^ These studies have all investigated the transmission of enteric pathogens across human, animal and environmental reservoirs and demonstrated the feasibility of integrated One Health approaches for mapping pathogen sources and assessing AMR. GETCampy builds directly on these foundational One Health studies but extends them in several ways: it utilises harmonised, multi-country genomic protocols across three West African countries, incorporates strain-level comparative genomics, and uses probabilistic and machine learning-based source attribution approaches to quantify the relative contributions of human, animal and environmental reservoirs. By doing so, GETCampy provides broader geographic coverage, standardised multisite methodology and analytical innovations, complementing previous single-country or single-cohort studies while generating actionable insights for public health interventions.

Africa remains underrepresented in global genomic surveillance and microbial evolution studies. This limits our understanding of regional transmission dynamics and skews global estimates of pathogen burden and AMR. It also constrains the development of tailored, region-specific interventions and policies. As international interest in African pathogen genomics is increasing,^54 55^ investigation efforts must be grounded in local priorities, with capacity-building integrated throughout any study. This will support long-term sustainability and equitable research partnerships. In this work, we present a standardised framework for the genomic epidemiology of enteric bacterial pathogens in LMICs. Our approach integrates One Health principles, ethical governance and local leadership. Using the GETCampy study as a case example, we demonstrate how harmonised protocols can be applied across diverse African settings to generate high-resolution data for source attribution, transmission mapping and targeted public health intervention.

## Design principles and methodologies

### Patient and public involvement

Prior to the start of the GETCampy-Africa study, researchers conducted site visits and stakeholder meetings with community leaders, health workers and public health officials in Burkina Faso, Ghana and The Gambia. These discussions informed the study design, including sample collection strategies, consent procedures and approaches to community engagement. In The Gambia, study participants were members of the general public attending routine care at MRCG health centres. Community input helped ensure that study materials and procedures were culturally appropriate and acceptable. Patients and the public were not directly involved in defining outcome measures or in the formal design of recruitment strategies. However, the study was developed in alignment with national public health priorities and frontline clinical experience in diarrhoeal disease management. Ongoing engagement with community stakeholders is planned to support interpretation and dissemination of study findings. Results will be shared through local stakeholder events, community workshops and accessible digital platforms, including the GETCampy-Africa website and YouTube channel.

### Ethics and dissemination

Ethical approval for this study was obtained from the Gambia Government/MRCG Joint Ethics Committee (Ref: 28260) and the London School of Hygiene and Tropical Medicine Research Ethics Committee (Ref: 28 260–01). Additional national approvals were obtained from the Ghana Health Service (Ref: GHS/NR/RHD/18/0/796) and the Tamale Teaching Hospital Ethical Review Committee (Ref: TTHERC/24/02/23/02). In Burkina Faso the study was approved by the Burkina Faso Health Research Ethics Committee (Ref: 2023‑06‑132).

Additional approvals for animal sampling were obtained from the National Agricultural Research Institute (The Gambia), the Faculty of Agriculture at the University for Development Studies and Pong-Tamale Animal Health and Production College (Ghana), and the Ministry of Agriculture, Animal Resources and Fisheries (Burkina Faso). Animal research was reviewed and approved by the LSHTM Animal Welfare and Ethical Review Board (Ref: 2025–06). Written informed consent was obtained from all participants or from parents/legal guardians of participating children prior to enrolment.

### Study objectives and outcomes

The overall objective of the GETCampy study is to apply a One Health genomic epidemiology framework to characterise the patterns of *Campylobacter* infections among children with medically attended diarrhoea (MAD) and to identify the relative contribution of human, animal and environmental reservoirs.

We hypothesise that *Campylobacter* prevalence is high among children under 5 years, children with MAD are more likely to carry strains genetically linked to human, animal or environmental reservoirs than matched asymptomatic controls, within-host *Campylobacter* genomic diversity is shaped by exposure to these reservoirs and AMR determinants and co-occurring enteric pathogens are more common among strains from clinical cases than from environmental or asymptomatic samples.

The primary outcomes of the study are the genomic relatedness and probabilistic assignment and association of *Campylobacter* isolates to MAD cases compared with isolates obtained from human, animal and environmental samples. These outcomes will be assessed using whole genome sequencing (WGS) and comparative genomic and machine learning–based source attribution approaches. Genomic relatedness will be quantified using pairwise single-nucleotide polymorphism (SNP) distances and phylogenetic clustering, while probabilistic source attribution will be estimated using machine learning and Bayesian assignment models. Secondary outcomes include the characterisation of within-host genomic diversity of *Campylobacter*, identification of AMR determinants and resistance profiles, and the detection of co-existing enteric pathogens. Within-host diversity will be measured using allele and SNP diversity metrics, AMR determinants will be identified through genomic screening against curated resistance gene databases, and co-occurring pathogens will be detected through both culture-based and metagenomic read-based analyses. These analytical approaches allow robust comparison of clinical, asymptomatic, animal and environmental samples.

WGS of cultured isolates will be used to quantify genomic relatedness among *Campylobacter* strains, including pairwise SNP distances and phylogenetic clustering. Metagenomic sequencing of stool and environmental samples will support the detection of co-existing enteric pathogens and assessment of within-host genetic diversity. Probabilistic source attribution and machine learning models will integrate WGS and metagenomic data to estimate reservoir contributions and likely sources of infection, ensuring that analyses align with the stated primary and secondary outcomes.

### Sample size and enrolment targets

GETCampy is a multisite genomic epidemiology and source attribution study rather than a hypothesis-driven clinical trial; therefore, formal statistical power calculations were not performed for the study. Target numbers were selected pragmatically to ensure adequate numbers of *Campylobacter* isolates per reservoir and site, rather than to detect predefined effect sizes or test specific hypotheses; numbers for each site and sample types are shown in table 1. Sample size and enrolment targets were prespecified in the protocol to ensure sufficient genomic diversity and representativeness across human, animal and environmental reservoirs to support comparative genomic and probabilistic source attribution analyses.

**Table 1:** Sample collection strategy by site

Subjects/environment	Expected samples by site			Total	Expected *Campylobacter* isolates by site*			*Campylobacter* isolates
					(isolation rate)			
	The Gambia	Ghana	**Burkina Faso**		The Gambia	Ghana	**Burkina Faso**	
Diarrhoea cases (stool/rectal swab)	300	600	520	1420	45 (15%)	90 (15%)	78 (15%)	213
Paired community control (stool)	135	–	–	135	4 (3%)	–	–	4
Household member (0–6 per *Campylobacter*-positive case) (stool)	Up to 270 (45*6)	–	–	Up to 270	8 (3%)	–	–	8
Animal reservoirs in cases’ household (stool)	Up to 180 (45*4)			Up to 180	54 (30%)			54
Environment samples from case’s household (food preparation, toilet area, water source, water storage)	Up to 225 (45*5)			Up to 225	11 (5%)			11
Environmental samples (effluents, grazing rivers, streams, sediment, abattoir runoffs)	Up to 100	Up to 100	Up to 100	Up to 300	5 (5%)	5 (5%)	Up to 5	15
Potential source reservoir: chicken/fowl (stool)	Up to 100	Up to 100	Up to 100	Up to 300	60 (60%)	60 (60%)	60 (60%)	Up to 180
Potential source reservoir: cow (stool)	Up to 100	Up to 100	Up to 100	Up to 300	20 (20%)	20 (20%)	20 (20%)	Up to 60
Potential source reservoir: horse/donkey (stool)	Up to 100	Up to 100	Up to 100	Up to 300	10 (10%)	10 (10%)	10 (10%)	Up to 30
Potential source reservoir: goat/sheep/pig (stool)	Up to 100	Up to 100	Up to 100	Up to 300	50 (50%)	50 (50%)	50 (50%)	Up to 150
Total	1610	1100	1020	3730	267	235	223	Up to 725

* Informed by previous studies.

Site-specific enrolment targets for Burkina Faso, Ghana and The Gambia were informed by expected healthcare attendance for MAD cases, anticipated *Campylobacter* positivity rates based on previous studies, laboratory capacity and the feasibility of simultaneous household and environmental sampling. Recruitment focused on children aged 0–59 months with MAD, alongside matched asymptomatic controls and asymptomatic household members, with simultaneous sampling of locally relevant household and community animal and environmental sources.

Enrolment was planned over a 2-year cross-sectional period at each site to capture seasonal variation, with ongoing monitoring to maintain balanced sampling across known reservoirs while accommodating local logistical constraints.

### Best practice guidelines for sampling enteric bacteria in Africa

**Figure 1:**
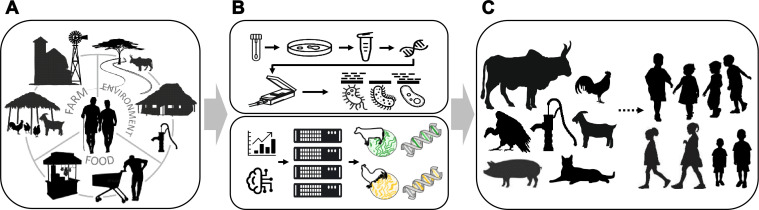
Enteropathogen (meta)genomic epidemiology for identifying infection reservoirs. (**A**) Human, animal, food and environmental reservoirs are sampled within a One Health framework. (**B**) Samples undergo culture-dependent and/or metagenomic analysis, followed by genomic sequencing and comparative genomic analyses to characterise pathogen populations and identify genetic variation associated with specific host reservoirs. Machine learning and statistical approaches are then used to develop source attribution models. (**C**) Source attribution models estimate the relative contribution of different reservoirs to human infection, enabling identification of major transmission pathways and infection reservoirs.

To maximise actionable inference in high burden settings, enteric pathogen studies benefit from structured survey designs that explicitly link human, animal and environmental sampling. For this study, a case-control design was selected because it allows comparison between children with MAD, asymptomatic controls and relevant household, animal and environmental sources. Depending on the study objectives, cross-sectional or longitudinal designs may also be used to capture population-level diversity across environmental, demographic and socio-economic settings.^56 57^ For *Campylobacter* and other enteric pathogens, case-control studies may provide greater insight into risk factors for MAD than infection or carriage alone, as previous studies, including GEMS and MAL-ED, have shown that pathogen detection correlates poorly with diarrhoeal symptoms in high-burden settings, where asymptomatic carriage is common. Study sites should therefore be selected to represent diverse ecological zones, urban and rural environments, and a range of cultural and agricultural practices (figure 1A).^58 59^

Local contextual knowledge is critical and, at each site, input from local researchers and stakeholders can identify culturally specific risk factors such as food preparation practices,^60^,^62^ waste-water management, common dietary exposures or livestock handling behaviours that may influence enteric pathogen transmission.^63^ Sampling strategies should account for variations in hygiene infrastructure, such as the absence of handwashing facilities or water treatment practices and include assessments of water, sanitation and hygiene (WASH)-related behaviours.^64^

Standardised protocols and quality assurance measures across all sites are, however, essential for comparability and regional relevance.^65^ Sampling designs should thus be sufficiently flexible to accommodate site-specific practices while maintaining harmonised methodologies for laboratory processing and data integration. The inclusion of local health authorities, community leaders and fieldworkers at the protocol design stage is very important to ensure the acceptability, deliverability and sustainability of study activities.

### Participant selection and environmental sampling strategy

The GETCampy study focuses on genomic differences and population structure between symptomatic and asymptomatic carriage. Household members and community control samples were included to provide depth of *Campylobacter* carriage and to provide comparator genomes for source attribution and population structure analyses. Controls were included to enable comparison of genomic lineages, diversity and inferred reservoirs between symptomatic and asymptomatic carriage within the same epidemiological context. All children 0–59 months presenting to GETCampy facilities with diarrhoea during working hours, whose caregivers consented were screened using a standardised questionnaire. Children living in the study catchment area and accompanied by a legal guardian were eligible. When more children were eligible than could be enrolled, a site-specific sampling strategy was applied to reach enrolment targets (~4–6 per week; ~25 per month in The Gambia), ensuring representativeness.

Participants at all sites met standardised criteria for inclusion. For this case study, individuals identified as having acute diarrhoea were matched with contextual comparators. Acute diarrhoea was defined as the passage of three or more loose or liquid stools per day, or more frequent passage than was normal for the individual, lasting less than 14 days (WHO).^64 66^ Applying this definition across all study sites and the inclusion of individuals experiencing acute diarrhoeal episodes and their contextual comparators: matched asymptomatic controls and asymptomatic household members ensured comparability.

To ensure reproducibility and minimise enumerator bias, sampling procedures were standardised across all participating sites and guided by harmonised standard operating procedures (SOPs), with strict adherence observed throughout study implementation. Sampling windows were defined relative to index case presentation, with enrolment and sampling of cases occurring on the day of presentation. Household, control, animal and environmental sampling were conducted within a 14-day window to ensure temporal alignment. Clear operational definitions were used for all human, animal and environmental sampling units, specifying exactly which individuals, animals and environmental sites were eligible for sampling, and where multiple eligible units were present, predefined, standardised protocol-driven selection rules were used. Field teams received harmonised and standardised training and used scripted data collection tools with embedded logic checks, supported by supervisory oversight to ensure continued compliance with SOPs. All procedures were clearly linked to shared and associated SOPs and centralised data dictionaries to ensure consistency across sites and over time.

Human, animal and environmental samples were collected according to a structured and standardised protocol to ensure reproducibility and minimise bias. Stool samples were obtained from children with MAD, their household members and age-matched and sex-matched community controls within 14 days of case presentation. Controls were selected based on compound proximity rules and eligibility criteria to avoid overmatching. Animal samples were collected from poultry, ruminants, pigs, horses and donkeys across study sites. Trained veterinarians and field staff observed animals until they defecated, collected fresh stool using sterile swabs, placed an aliquot in Cary Blair transport medium for bacteriological culture, and preserved the remainder for metagenomic analysis. Environmental samples, including water, sediment and household surfaces, were collected systematically from wells, streams, slaughterhouses and household food preparation areas using sterile techniques; electrostatic cloths were used specifically for household surface samples. All field staff were trained on the sample collection SOPs, including timing, sample handling, storage and transportation to the laboratory, to ensure consistency across sites and reduce potential enumerator bias. SOPs specified the order and timing of sample collection relative to events such as handwashing, feeding or animal defecation to minimise bias in pathogen detection across enumerators and sites. Detailed sampling plans, including numbers and types of expected samples, are shown in table 1.

Eligibility criteria for cases were explicitly defined to ensure reproducibility. Inclusion criteria for cases included children aged 0–59 months who presented to GETCampy with MAD, defined as three or more loose or watery stools in 24 hours, with or without blood, requiring medical attention at participating facilities. Children were required to reside within the pre-defined study area, and the diarrhoeal episode had to be acute, with onset within 7 days of study enrolment, and represent a new episode following at least seven diarrhoea-free days. Caregivers were required to provide informed consent in the local language and be willing to have stool samples or rectal swabs collected from the child at enrolment. Children were excluded if they were being referred to a non-GETCampy facility at screening. Controls consisted of age-matched asymptomatic household members and community children who had not experienced diarrhoea in the preceding 14 days.

Eligibility criteria for community controls in The Gambia were explicitly defined for comparability and reproducibility. Controls were matched to the corresponding index MAD case by age, gender and village/community. Enrolment was conducted within 14 days of presentation of the index case. Controls were required to be diarrhoea-free within the 7 days preceding enrolment. Primary caregivers had to be able to provide informed consent in the local language and be willing for samples to be collected from their child at enrolment. If no eligible control or household member was available due to recent diarrhoea or refusal, a matched community child was selected as the comparator. In rare cases where no suitable control could be identified, the corresponding MAD case remained enrolled but was flagged for analysis as unmatched, ensuring transparency in data interpretation.

Exclusion criteria included children who were outside the eligible age range (0–59 months), resided outside the predefined study area, had severe comorbidities or were enrolled in another study. Children were also excluded if their primary caregiver did not provide informed consent, was unwilling to provide stool samples, currently enrolled in the GETCampy study as a case or control (defined as enrolment within the preceding 60 days), or unable to provide a sufficient stool sample, defined as less than 4 grams (approximately the size of four peas) within the allowable collection period.

Other relevant samples were also systematically collected. These included other human stool samples (cross-sectional sampling), drinking water, swabs from food preparation areas, soil, animal faeces and market food items. Stratified sampling was deliberately used to ensure adequate representation of demographic groups. Stakeholder and participant engagement was embedded in recruitment procedures to foster trust and facilitate participation.

### Enrolment and sampling timeline

To ensure consistency and reproducibility across sites, the enrolment and sampling timeline was clearly defined. Children with MAD presenting to GETCampy recruitment health facilities were screened for eligibility and enrolled on the day of clinic presentation. Collection of stool or rectal swab samples occurred at enrolment. Children with MAD were enrolled and sampled on the day of clinic presentation, before laboratory confirmation of *Campylobacter* infection. Household and community controls were then recruited within 14 days of the index case visit, once the MAD case’s stool sample had been confirmed as *Campylobacter*-positive. This workflow allowed for timely enrolment while ensuring that only confirmed cases guided control and household selection. Household follow-up for matched controls and environmental sampling, including stool from domestic and farm animals, water and surface swabs, was conducted within 14 days of the index case presentation and confirmation of *Campylobacter* positivity of the corresponding MAD case. All sampling activities were coordinated to occur within 14 days of the index case clinic visit, ensuring temporal alignment of human, animal, and environmental data. This standardised timeline allowed for robust source attribution analyses and comparison across study sites.

### Data management

Standardisation of data collection tools and procedures is essential to ensure consistency within and across sites. Secure database platforms such as REDCap^67^ are widely used for epidemiological data capture, while other open-source resources including DataFlow^68^ and PubMLST^69 70^ can support harmonised storage of microbiological and genomic data. Together, these platforms enable linkage of participant metadata, sample tracking, microbiology results and genomic data across study phases. Integrated database systems support real-time data entry and sharing between field, laboratory and analyses teams, and improve the traceability of samples throughout the project lifecycle.

Clear operational definitions for all variables and SOPs should be developed collaboratively and shared across all participating sites. Field and laboratory staff must be trained using unified curricula, with refresher training and inter-laboratory benchmarking implemented regularly to minimise procedural drift. Centralised data dictionaries and template forms can support harmonised data reporting and improve data quality.

### Data availability

This manuscript describes study design, protocols and analytical frameworks only. No datasets were generated or analysed as part of this work. Participant enrolment and sample collection have been completed, while laboratory processing, sequencing and data analysis are ongoing. Data generated from the GETCampy study will be governed by local ethical approvals, national regulations and consortium data-sharing agreements, and will be made available in appropriate public repositories or on reasonable request in future outcome-focused publications.

### Microbiological methods

For reliable comparability among sites and over time, samples must be processed promptly using standardised microbiological methods to isolate enteric pathogens, including but not limited to *Campylobacter*, *Shigella*, *Salmonella* and pathogenic *E. coli*. Technical SOPs should be developed collaboratively and disseminated across sites to ensure consistency. Cross-site training programmes, including hands-on workshops and remote support, should be implemented to align technical practices and build staff capacity. External Quality Assessment (EQA) schemes should be used to evaluate and maintain laboratory performance.^71^,^73^

Pragmatic flexibility is also essential. Protocols should be adaptable to accommodate local constraints in lower-resource settings, such as limited availability of reagents, equipment or cold chain infrastructure. Planning and responsive decisions on diagnostic workflows should balance standardisation with feasibility, leveraging available infrastructure while maintaining data quality. For technical staff, a strong foundation in microbiology is vital; laboratory personnel should possess demonstrated experience in enteric pathogen isolation, culture techniques, and quality control.

### Sequencing and bioinformatic analysis

While it is ideal to build genomic sequencing capacity and infrastructure locally, challenges often arise with access to reagents and computational resources in low-resource settings. Oxford Nanopore Technologies (ONT) platforms offer logistical advantages due to their portability and lower initial capital outlay.^74^,^76^ However, sustainable capacity-building must go beyond instrument access and include the empowerment of local researchers to conduct their own analyses. To support this, cloud-based and publicly accessible genomics platforms such as PubMLST,^69 70^ Pathogenwatch^77^ and EnteroBase^78 79^ provide user-friendly tools for sequence analysis, strain typing and AMR profiling. These platforms reduce reliance on local bioinformatic infrastructure and enable researchers to engage with their data independently while aligning with global standards.

For genomic epidemiology studies, the first and primary consideration is the generation of sufficient quantities of pure uncontaminated DNA from samples. High-quality genomic data can be generated using next-generation sequencing technologies such as Oxford Nanopore and Illumina platforms. Combining long-read and short-read sequencing can also be beneficial, allowing for comprehensive genomic characterisation, including identification of AMR genes, virulence factors and strain-level differentiation (figure 1B). Sequencing of bacterial isolates sub-cultured in the lab from primary faecal samples can follow standard plate subsampling of single colonies and extraction protocols and is the basis of large-scale enteric pathogen studies.^80^,^83^ Sweep-metagenomics can also be used to simultaneously sequence multiple enteric bacteria cultured on a single plate.^84^ Direct metagenomic sequencing from primary samples may be preferred. This can be the case where rapid diagnostics are needed without waiting for culture or where samples of sufficient quality for isolate sub-culturing cannot be stored or rapidly conveyed to a laboratory, for example in remote low-resource settings. Enteric pathogen metagenomic surveillance has been conducted on wastewater and patient faecal samples in Africa.^85^,^88^

### Analysis framework

Genomic similarity among *Campylobacter* isolates from children with MAD, household members, community controls, domestic and farm animals, and environmental samples will be interpreted as evidence of shared reservoirs or exposure pathways. Such similarity may reflect common environmental exposure, asymptomatic carriage or onward shedding between individuals or into the environment and was not interpreted as evidence of direct or directional transmission. Probabilistic source attribution models, including machine learning approaches, will help estimate the likely contributions of different reservoirs to observed human infections, accounting for the temporal alignment of sampling and cross-site population diversity. These models quantify population-level associations and relative reservoir contributions rather than identifying the origin of infection for individual cases. Analyses will be conducted using harmonised bioinformatics pipelines, with outputs interpreted in the context of probability and association. Model results will be linked to risk factor and contextual metadata where appropriate to assess patterns of exposure across the human-animal-environment interface.

### Quantifying pathogen transmission dynamics and targeting interventions

The reason for pathogen surveillance is to inform interventions to reduce infection. For pathogens circulating in high burden settings, this requires quantification of the relative importance of different reservoirs of infection. Like all organisms, enteric pathogens adapt to their host environment, in this case the gut. These adaptations are reflected in the pathogen genome.^33 83^ Sequencing the genome of strains from humans, reservoir hosts and faecal contaminated environments allows the identification of genomic host-segregating markers (genes, alleles, SNPs).^89^ These markers can be used in probabilistic and machine learning models to quantitatively assign where clinical cases originated (figure 1B).^3383 90^,^92^ Data on the relative contribution of different pathogen reservoirs to human disease can be used for risk calculation and to inform targeted interventions (figure 1C). For example, by calculating the impact of potential interventions on disability-adjusted life-years, decision support tools can provide a monetised cost-breakdown of the most effective intervention points in the transmission network, essential for policymakers.

### Project management

In addition to the collaborative design and execution of the technical components, it is important to maintain strong within-project confidence and communication and enable equitable knowledge sharing. These are achieved through regular, well-attended meetings including key senior and junior staff. Practically, these are held online, though in-person meetings may be recommended during the life of the project. External communication of project outcomes should also form a part of the overall study design whether in the form of academic papers, technical reports or conference presentations. Iterative and transparent development of co-authored academic papers allows all researchers to participate and can model manuscript development, a vital part of the training of young academics in any country and of researcher capacity building.

### Case study: GETCampy

**Figure 2:**
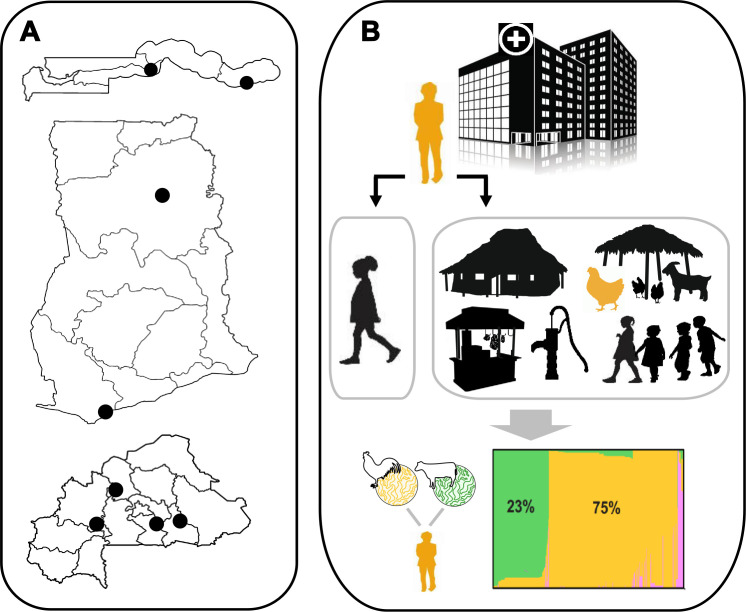
*C*ase study design for *Campylobacter* genomic epidemiology. (**A**) Sampling network in healthcare centres (●) in (1) The Gambia—Yurrobawol Health Centre and Basse District Hospital; (2) Ghana—Tamale Teaching Hospital and Ekkia Nkwanta Hospital; (3) Burkina Faso—Boromo, Manga, Tenkodogo and Yako Health Centres. (**B**) Sampling structure to identify the source of positive cases of *Campylobacter* medically attended diarrhoea. Direct comparison is made to (1) household members (controls) and (2) high-contact domestic and farm animals, household surfaces, water sources and other human and environmental samples. All (1) case–control and (2) cross-sectional samples are DNA sequenced to enable (3) quantitative source attribution using probabilistic and machine learning models, to determine the relative contribution of different disease sources. In this example, human isolates (columns) are attributed to source reservoirs (chicken-yellow; cattle-green).

Following the principles outlined above, the GETCampy project was conducted with partners in four countries: Burkina Faso, Ghana, The Gambia and the UK (figure 2). The study was conducted between August 2023 and June 2024, during which participant enrolment and sample collection were completed; laboratory processing and genomic analyses are ongoing. The 2-year cross-sectional multisite study enrolled children aged 0–59 months presenting with MAD, matched asymptomatic family members and community controls at sites in three countries (figure 2A).

MAD cases were identified at the clinic, with caregivers providing consent, following which stool/rectal swabs were collected immediately. *Campylobacter* diagnosis was completed within 48–72 hours, after which age-matched, sex-matched and community-matched controls and household members were enrolled within 14 days. Cases were enrolled consecutively during clinic hours (~4–6 per week per site), and sample sizes were selected pragmatically to ensure sufficient *Campylobacter* isolates per site and reservoir type. Additional environmental samples collected included stool from humans and animals, swabs from household surfaces and food preparation areas, and local water samples. Eligibility criteria included age 0–59 months, residence within the study area, a new diarrhoeal episode within 7 days, and caregiver consent; controls had to be diarrhoea-free for 7 days, with similar residence and age criteria. Exclusion criteria included severe comorbidities, enrolment in another study or inability to provide sufficient stool.

*Campylobacter* isolates were cultured and identified using standardised microbiological techniques and whole faecal samples were also collected. All cultured isolates underwent hybrid sequencing using both Oxford Nanopore (long-read) and Illumina (short-read) sequencing technologies, facilitating high-quality genomic analyses and whole sample DNA extracts were metagenome sequenced. Genomic data were analysed using standardised bioinformatics pipelines. Comparative genomic analyses identified genetic diversity, population structure, and probabilistic source attribution. The structured timeline of clinic enrolment, diagnosis, household visits and control recruitment ensured temporal alignment of samples for robust source attribution. Advanced machine learning methods were applied for robust source attribution, estimating the likely contributions of human, animal and environmental reservoirs and associated exposure patterns. Quantitative data describing source-sink dynamics informed decision support models to identify the optimal targets for interventions, supporting context-specific public health strategies designed to significantly reduce diarrhoeal disease burdens and improve child health in LMICs. Detailed methods, SOPs and consenting protocols are provided in the Supplementary Information.

## Discussion

This design framework and associated protocol present a structured and scalable approach to exploring the genomic epidemiology of enteric bacterial pathogens across Africa. Sampling and analyses integrate environmental, clinical and genomic data, and the GETCampy study exemplifies the application of this framework, combining cross-sectoral sampling, community engagement and high-resolution WGS to trace probable exposure pathways and inform potentially targeted interventions. The strength of this case study comes from the One Health design, which incorporates multiple bacterial species and uses robust analytical methods, including machine learning and comparative genomics. By embedding the study within existing public health and research infrastructure across Burkina Faso, Ghana and The Gambia, GETCampy demonstrates both feasibility and relevance for other LMIC settings. Our framework complements and extends prior One Health enteric research in Africa. While studies such as CAGED, PATHOME and Urban Zoo helped provide valuable insights into local transmission dynamics and reservoir contributions, the GETCampy study design expands these approaches across multiple countries, integrates high-resolution strain-level genomics and applies probabilistic and machine learning-based source attribution methods. Together, these elements provide a robust platform for identifying likely exposure pathways and supporting decisions about intervention priorities where evidence suggests likely reservoir contributions without relying on outcome reporting.

The under-representation of the diverse ecological and socio-behavioural contexts in Africa hampers collective understanding of pathogen diversity, transmission and the spread of AMR. Ethically informed and collaboratively designed studies of African pathogen genomics contribute critically to global scientific knowledge, but also to equity, public health relevance and global pandemic preparedness. Pathogens do not respect borders, intercontinental trade, migration and zoonotic transmission networks demand that global analyses account for African data if we are to develop effective vaccines, diagnostics and public health interventions.

By applying our principles and sharing the case study experience and suggestions for study design and protocol implementation, we have addressed four important elements. First, the integrated One Health design identifies sources of exposure linked to humans, animals and environmental sources. Second, local capacity-building and the use of portable sequencing platforms and open-access analytic tools promote sustainable genomics for public health. Third, cross-site harmonisation, supported by shared SOPs, training materials and EQA schemes builds collaboration and the knowledge economy in the region. Finally, ethical and equitable governance, with local leadership and Nagoya Protocol adherence, increases the likelihood of long-term benefit sharing among the people at greatest need.

Despite careful planning and adherence to key principles, logistical and operational challenges inevitably arise. Laboratory capacity and supply chains can be unpredictable, requiring protocol flexibility. Training field and laboratory staff to a consistent standard across multiple sites is resource-intensive and without regular support local norms can re-emerge. Furthermore, retention of expertise is difficult in resource-limited settings. Despite the use of accessible tools (eg, Pathogen.watch, PubMLST), bioinformatic analysis and data interpretation remain skill-intensive and often depend on stable internet access. Finally, standardising community engagement across sites remains complex and must be tailored to cultural context. Ethical oversight and governance must go beyond institutional approvals to include ongoing monitoring, transparency and fair representation of local voices in governance and authorship.^93^

Our study has several limitations. First, the cross-sectional and case–control sampling framework we employed does not support inference about the directionality of transmission between humans, animals or environmental reservoirs. While probabilistic source attribution and genomic similarity analyses provide insight into likely shared reservoirs or exposure pathways, they cannot definitively establish the source and direction of transmission. Second, as samples from household members, community controls and environmental sources were collected within a predefined 14-day window rather than simultaneously with cases, which introduces temporal ambiguity. Finally, potential confounding by uncontrolled behavioural, environmental or host factors cannot be excluded and should be considered when interpreting observed associations. Together, our limitations highlight that results from this study will reflect patterns of exposure and likely contributions of reservoirs, rather than establishing direct causal transmission.

In this context, GETCampy should be interpreted as providing population-level genomic evidence of likely exposure pathways rather than direct transmission events. When considered alongside longitudinal or setting-specific One Health studies such as CAGED, PATHOME and Urban Zoo, which examine finer-scale transmission dynamics, GETCampy contributes harmonised, multicountry strain-level data that can help prioritise likely reservoirs and targeted intervention across diverse resource-limited African settings.

In conclusion, these data have the potential to support the design of more targeted interventions based on observed exposure patterns, including WASH improvements, antimicrobial stewardship policies and potentially vaccine development. The falling cost of genome sequencing has the potential for the implementation of genomic epidemiology in LMICs. In the long term, this may be applied for longitudinal surveillance, real-time outbreak response, and could even be integrated into national public health strategies, where needed. While global funding sources can boost this process, regional pathogen genomic networks should be driven and governed by African institutions, supported by sustainable funding and embedded within national policy-making bodies. By supporting equitable partnerships, open science and ethical governance principles, enteric genomics studies can contribute meaningfully to both local health improvements and global pathogen surveillance.
