# Supplementary material for: Protocols for genomic epidemiology and source attribution of enteric bacteria causing diarrhoeal disease across Africa
Source: BMJ Open. 2026 Jun 25;16(6):e109566. doi: 10.1136/bmjopen-2025-109566 (PMC13311725; doi:10.1136/bmjopen-2025-109566)
Supplement: Supplementary file 1 [file bmjopen-2025-109566.reviewer_comments.pdf]

PEER REVIEW HISTORY

BMJ Open publishes all reviews undertaken for accepted manuscripts. Reviewers are asked to complete a checklist review form and are provided with free text boxes to elaborate on their assessment. These free text comments are reproduced below.

ARTICLE DETAILS

Title (Provisional)

Protocols for genomic epidemiology and source attribution of enteric bacteria causing diarrhoeal disease across Africa

Authors

Badji, Henry; Pascoe, Ben; Goforth, Madison P; Mourkas, Evangelos; Bako, Evariste; Bonkoungou, Isidore J; Gampene, Modeste T; Nikiema, Marguerite EM; Zoma, Barthélemy S; Bah, Ousman E; Cassell, Eustacia J; Ceesay, Abdoulie K; Ceesay, Bubacarr E; Ceesay, Ousman; Conteh, Bakary; Drammeh, Lamin; Faye, Binta; Jallow, Abdoulie F; Jallow, Fatima; Jallow, Ousman; Jallow, Samba J; Joof, Pa M; Kandeh, Modou; Karim, Mehrab; Manneh, Jarra; Ceesay, Abdou; Fofana, Ebrima; Jallow, Mamud; Jarju, Modou B; Bah, Samba; Sanyang, Dodou; Jallow, Demba B; Alebiosu, Mukaila I; Brusah, Atanyiwoen; Karikari, Akosua Bonsu; Saba, Courage KS; Haukka, Kaisa; Collins, C Matilda (Tilly); Leach, Adrian W; Levontin, Polina; Ali, Shani UP; Colles, Frances M; Hitchings, Matthew David; Antonio, Martin; Sesay, Abdul K; Gundogdu, Ozan; Hossain, M Jahangir; Sheppard, Samuel K

VERSION 1 - REVIEW

**Reviewer**	**1**
**Name**	**Baker, Kelly**
**Affiliation**	**University of Iowa, Department of Occupational and Environmental Health**
**Date**	**01-Dec-2025**
**COI**	**None**

Review of Badji et al “Protocols for genomic epidemiology and source attribution of enteric bacteria causing diarrhoeal disease across Africa”

The GetCampy study is using a case-control design and genomic analysis of Campylobacter isolates, perhaps whole feces samples as well, to identify other potential human, animal, and environmental sources of Campylobacter and examine their base pair relatedness to infected cases with medically attended diarrhea. The study could build upon an existing body of studies on transmission of enteric

pathogens, including Campylobacter, in Africa. Protocols of studies using one health approaches to understand enteric disease transmission are needed to help other researchers adopt similar strategis in their studies. The whole genome sequencing approach is well suited for identifying within-species differences in genes, gene fragments, plasmids, or mutations that could improve confidence in linkages between a case and their environment. While the microbiology protocols are rigorous, the epidemiological detail and language used does not clearly describe how the study design, methods, and analysis will be used to address study goals.

MAJOR COMMENTS

It is not clear from the manuscript whether this is a planned or ongoing study versus a concluded one as no dates are reported in the abstract or study design section. The abstract uses "were" with reference to enrollment and data collection and there is a takeaway bullet point and comment in the discussion referring to lessons learned that suggest the study is done. Yet lines 433-436 state no data has been collected. The dates need to be stated in the abstract and study design section to better demonstrate compliance with journal guidelines requiring protocols describe planned or ongoing studies.

The authors include a 2013 Spirit Checklist that suggests core study details for an observational epidemiological study were defined a priori. However, details about specific objectives and hypotheses, analytical outcomes to be measured/modeled, sample size, eligibility criteria, and enrollment timeline are not provided as is expected in an epidemiological protocol. Thus, a reader would not be able to replicate this study. Specific points where missing data should be provided are detailed below in itemized comments.

The language throughout the manuscript describes a study that uses bioinformatics and machine learning algorithms with human, animal, and environmental data to quantify pathogen diversity, sources of infection, and transmission pathways. The microbiological protocols are suitable for measuring pathogen diversity, although when and how metagenomic versus whole genome data will be used to address what study analysis is not described. Clear articulation of primary and secondary study outcomes that will be used to quantify and compare diversity, identify sources, and identify transmission pathways (e.g. base pair or Alpha and beta diversity?) is needed for future confirmation that future analysis is aligned with original stated purpose. It would also clarify whether this is a comparative study of Campylobacter WGS or taxonomy.

The authors may be overstating the capacity of this study design to identify pathogen sources and inform interventions on transmission pathways although further detail on analytical approach may resolve this concern. High quality genomic data and bioinformatics tools are powerful tools for quantifying relatedness/associations

between index cases and potential sources or pathways of transmission, but if data is collected using a study design that does not support causal inference, then association evidence only indicates potential for bidirectional pathogen transmission, not sources and directionality of pathogen flow. As noted in detailed comments below, the temporal design of the case-control study means that there is an equal chance the study will measure the risk that the case poses for bacterial transmission TO household members, animals and environment as the risk FROM household members, animals, and environment to the case. Authors should be clearer about how comparative genomics relatedness evidence will be interpreted in methods and justify how the analytic approach will support causal inference about pathogen sources (lines 53-54, 119, 246-249, 346) given the study design.

The microbiological rigor and detail and consideration of data pipelines however is strong.

The inclusion of language about partnership ethos and teamwork approach is appreciated.

DETAILED COMMENTS

Page 4 lines 99-101 The line "These studies, however, primarily relied on conventional culture or molecular diagnostics, and lacked the genomic resolution needed to investigate pathogen diversity, transmission pathways, and antimicrobial resistance (AMR) [50]" is scientifically incorrect in its assumption that only genomics can address these research questions. Multi-pathogen culture and molecular approaches can provide disease-constrained measures of microbial community species richness and diversity at genus, species and even sero/pathotype levels, as well as AMR pheno/genotypes. A WGS genomic approach provides a deeper base pair level capacity for detecting differences within individual species, but that does not negate higher order taxonomic methods for source and transmission mapping. Metagenomic sequencing allows one to use non-pathogenic microbes for pattern recognition, but is not necessary for disease ecologies (like enteric infections) where species richness within endemic communities is high.

Page 4 lines 96-106: There are several large One Health studies on enteric pathogen diversity, sources, and transmission dynamics in Africa that predate this one and should be referenced and discussed as far as how this study complements and differs. Please reference and discuss the CAGED study in Ethiopia and PATHOME and Urban Zoo studies in Kenya (protocols that link to their other papers below) in particular. A quick pubmed search revealed multiple another recent studies that may be informative for describing value added from this new study.

- Havelaar AH, Brhane M, Ahmed IA, Kedir J, Chen D, Deblais L, French N, Gebreyes WA, Hassen JY, Li X, Manary MJ, Mekuria Z, Ibrahim AM, Mummed B,

Ojeda A, Rajashekara G, Roba KT, Saleem C, Singh N, Usmane IA, Yang Y, Yimer G, McKune S. Unravelling the reservoirs for colonisation of infants with *Campylobacter* spp. in rural Ethiopia: protocol for a longitudinal study during a global pandemic and political tensions. BMJ Open. 2022 Oct 5;12(10):e061311. doi: 10.1136/bmjopen-2022-061311. PMID: 36198455; PMCID: PMC9535169.

- Baker KK, Simiyu S, Busienei P, Gutema FD, Okoth B, Agira J, Amondi CS, Ziraba A, Kapanka AG, Osinuga A, Ouma C, Sewell DK, Gaire S, Tumwebaze IK, Mberu B. Protocol for the PATHOME study: a cohort study on urban societal development and the ecology of enteric disease transmission among infants, domestic animals and the environment. BMJ Open. 2023 Nov 24;13(11):e076067. doi: 10.1136/bmjopen-2023-076067. PMID: 38000826; PMCID: PMC10680014.
- Hassell JM, Muloi DM, VanderWaal KL, Ward MJ, Bettridge J, Gitahi N, Ouko T, Imboma T, Akoko J, Karani M, Muinde P, Nakamura Y, Alumasa L, Furmaga E, Kaitho T, Amanya F, Ogendo A, Fava F, Wee BA, Phan H, Kiiru J, Kang'ethe E, Kariuki S, Robinson T, Begon M, Woolhouse MEJ, Fèvre EM. Epidemiological connectivity between humans and animals across an urban landscape. Proc Natl Acad Sci U S A. 2023 Jul 18;120(29):e2218860120. doi: 10.1073/pnas.2218860120. Epub 2023 Jul 14. PMID: 37450494; PMCID: PMC10629570.

Page 6 lines 139 to 142: This section uses the words “the optimal study design” and then proceeds to name the three most common types of epidemiological designs, suggesting all epi design approaches are optimal designs. Please remove or make the point of this statement clearer.

Page 6 lines 141-142 the primary subject identification procedure uses a case-control design with Medically attended diarrhea (MAD) as the criterion for case or control classification. Many studies, including the GEMS and MAL-ED studies cited in this manuscript and many others, have highlighted poor correlation between diarrhea and detection of pathogens in kids in high burden settings and high asymptomatic burden (mentioned on lines 84-85). Based upon those studies, enrolling a control based upon a lack of diarrhea symptoms gives the study team an approximately 25 to 40% chance of enrolling a control without some type of a subacute infection, depending upon subject age and geographic location. Many mild diarrhea cases or cases that do not meet WHO criteria (e.g. diarrhea twice in 24 hours) do not go to clinic and would be classified as a health control in this study. Therefore, these lines should be revised to state the case-control design provides valuable information about MAD risk factors, not infection risk factors.

Page 6 lines 150 to 152: This suggests that there was a systematic sampling plan that would advise field staff on when and how to collect human feces, animal feces, or environmental samples. Standardized protocols are critical for ensuring enumerator

beliefs and attitudes about sample collection do not bias the chances of detecting different types of bacteria, e.g., random hand rinses vs 5 minutes after handwashing. There are no details provided about how sampling was done.

Pages 7 to 11 Lines 170 to 340: The entire structure of this section does not flow clearly. Further, it does not present sufficient information that would let anyone repeat the study or understand what was done.

- There is no sample size section describing the intended number of subjects nor how that number was calculated.
- There is a paragraph 170-181 that is entitled "Participant selection and environmental sampling strategy" which refers to a case-control study, as in the introduction, but it provides no detail about the study design process. Health care centers are mentioned in the legend of Figure 2 so presumably recruitment and case enrollment happens there. Yet how enrollment was done and what eligibility and exclusion criteria were is not described which makes it problematic to understand who is actually considered a case. Cases are described as children with MAD, but health controls in the household are controls for Campylobacter positive MAD patients, implying there is a diagnosis process occurring between a MAD case presenting at clinic and confirmation that controls should be recruited. When and where is this Campy diagnosis happening? Does the case remain at the clinic until diagnosis is done (Fig 2 suggests enrollment is done at clinic) or does the case go home with household follow up being dailyed for some period of time until Campy diagnosis is complete? How long does the whole process take between diagnosis, case enrollment, control enrollment, and data collection?
- Did clinics enroll all cases, a proportion of cases, a number of cases per time interval, etcetera?
- Child age appears to be the only case eligibility criteria. Please confirm. No eligibility criteria are described for controls.
- The title "OBSERVATIONAL STUDY: genomic epidemiology and transmission of Campylobacter (GETCampy)" is inconsistently formatted relative to other sections. It also does not contain complete information and references a cross-sectional follow-up, which seems to simply be completion of the control enrollment/data collection part of the case-control study. The lack of a study timeline means that it is unclear when household visits and control enrollment happen relative to case identification nor are there details on how controls are selected and enrolled. Is the team enrolling everyone in the household as a control or picking one member as a representative control? What happens when all eligible controls can't be controls because they just had diarrhea? Would there be no control and thus the case child is disenrolled? How long are cases and controls enrolled in the study?

- Protocols for epidemiological studies are expected to report enough detail about the study design for public assessment of rigor, reproducibility, and transparency. Please reorganize and map paragraph content against the SPIRIT guidelines that were chosen as a reporting tool.

Page 10 Line 281-283 - This has nothing to do with the study approach. Please move to introduction or discussion or revise to make the approach detail apparent.

Please create a distinct Analysis section that describes how each type of genomic evidence will be used to address objectives (diversity, pathogen sources, transmission pathways). Per the big picture comments above, either revise language throughout to remove suggestion of causal inference or clarify how bioinformatics approaches will use case-control(s) study design data to make conclusions about pathogen sources and pathways. With the described design, it is just as likely that the case shed a strain of Campylobacter (or other pathogens) in the household and infected the control(s), who could be asymptomatic but still shedding pathogens. The same question about directionality arises as far as whether the case is the victim or source of pathogens deteted in the environment and animals.

Page 12 – The discussion should include a paragraph on limitations of the described study approach. Per comments about pre-existing CAGED/PATHOME/Urban Zoo pathogen transmission studies, how might the evidence from GetCampy be used alongside or address gaps in those studies to address the mutual goal of these studies to improve the use of effective disease control interventions?

Page 106 - There is a data dictionary for a survey in the appendix, but no reference to when or how it is used in the recruitment, enrollment, and matriculation process.

FORMATTING COMMENTS:

A simple grammar review would be beneficial as there are some nonsensical statements, like "it is recommended that enteric bacterial pathogens should use a structured survey approach (line 137-138)" and "pathogens circulating in one health settings (lines 241-242). Bacteria cannot conduct surveys and all places are one health settings so what is the counterfactual to this? Do the authors mean high disease burden settings?

Many of the pictures in the SOP are poor quality resolution and are not cited suggesting these are original pictures of bacteria plates (Fig 2) and stains and tests (Figs 4-8). All images replicated from standard microbiology reference guides (FDA BAM or EU EFSA or textbooks) should have credits as to source of the image and reuse approval.

VERSION 1 - AUTHOR RESPONSE

Reviewers' comments

MAJOR COMMENTS

1. Study status (planned vs ongoing vs completed) is unclear

Reviewer comment: It is not clear from the manuscript whether this is a planned or ongoing study versus a concluded one as no dates are reported in the abstract or study design section. The abstract uses "were" with reference to enrollment and data collection and there is a takeaway bullet point and comment in the discussion referring to lessons learned that suggest the study is done. Yet lines 433-436 state no data has been collected. The dates need to be stated in the abstract and study design section to better demonstrate compliance with journal guidelines requiring protocols describe planned or ongoing studies.

Response: *We thank the reviewer for identifying this important ambiguity. We clarify that participant enrolment and sample collection for the GETCampy study have been completed, while laboratory processing, sequencing, and genomic analyses are ongoing. This manuscript presents the study design, protocols, and analytical framework and does not report outcome data.*

To address this, we have:

- *Revised the Abstract and Study design sections to explicitly state that sampling has been completed and analyses are ongoing (**Abstract, line 47-48**).*
- *Standardised verb tense throughout the manuscript.*
- *Added explicit clarification that no outcome data are reported in this protocol manuscript.*
- *Updated the Data Availability Statement to reflect that no datasets were generated or analysed for this manuscript (**data availability statement, lines 352-358**).*

Together, these revisions provide sufficient detail for independent assessment, transparency, and replication of the study design.

2. Insufficient epidemiological detail to support reproducibility

Reviewer comment: The authors include a 2013 Spirit Checklist that suggests core study details for an observational epidemiological study were defined a priori. However, details about specific objectives and hypotheses, analytical outcomes to be measured/modelled, sample size, eligibility criteria, and enrolment timeline are not provided as is expected in an epidemiological protocol. Thus, a reader would not be able to replicate this study. Specific points where missing data should be provided are detailed below in itemized comments.

Response: *We agree and thank the reviewer for this detailed and constructive assessment. In response, we have substantially strengthened the epidemiological description of the study and explicitly aligned the Methods with SPIRIT 2013 guidance.*

*Specifically, we have revised the manuscript to clearly define the study objectives and outcomes (**lines 156-168**), eligibility criteria for cases and controls (**lines 248-254, 286-313**), enrolment procedures and timelines, and intended sample size targets across sites (**lines 321-335**). We have also clarified the analytical framework used to address diversity, attribution, and transmission questions (**lines 402-415**), ensuring that the protocol can be independently assessed and replicated.*

Together, these revisions bring the manuscript into full alignment with SPIRIT reporting

expectations for observational study protocols.

Reviewer comment: Clear articulation of primary and secondary study outcomes that will be used to quantify and compare diversity, identify sources, and identify transmission pathways (e.g. base pair or Alpha and beta diversity?) is needed for future confirmation that future analysis is aligned with original stated purpose. It would also clarify whether this is a comparative study of Campylobacter WGS or taxonomy.

*We have added a **Study objectives and outcomes** subsection (**Lines 156-168**) defining:*

- ***Primary outcomes:** genomic relatedness and probabilistic attribution of Campylobacter isolates between cases and contemporaneous human, animal, and environmental samples.*
- ***Secondary outcomes:** within-host diversity, antimicrobial resistance profiles, and co-occurring enteric pathogens.*

In addition, we have:

- *Added a Sample size and enrolment targets subsection (Lines 177-185) describing intended recruitment numbers per site and pragmatic considerations informing these targets.*
- *Explicitly defined case and control eligibility criteria, including age range, clinical definition of medically attended diarrhoea, and exclusion criteria (Lines 286-313).*
- *Clarified the enrolment and sampling timeline, including clinic presentation, diagnostic confirmation, household follow-up, and environmental sampling windows (Lines 321-335).*

These revisions provide sufficient detail for independent assessment and replication of the study design.

3. Overstatement of genomics relative to conventional microbiological approaches

Reviewer comment: The authors may be overstating the capacity of this study design to identify pathogen sources and inform interventions on transmission pathways although further detail on analytical approach may resolve this concern. High quality genomic data and bioinformatics tools are powerful tools for quantifying relatedness/associations between index cases and potential sources or pathways of transmission, but if data is collected using a study design that does not support causal inference, then association evidence only indicates potential for bidirectional pathogen transmission, not sources and directionality of pathogen flow. As noted in detailed comments below, the temporal design of the case-control study means that there is an equal chance the study will measure the risk that the case poses for bacterial transmission TO household members, animals and environment as the risk FROM household members, animals, and environment to the case. Authors should be clearer about how comparative genomics relatedness evidence will be interpreted in methods and justify how the analytic approach will support causal inference about pathogen sources (lines 53-54,119, 246-249, 346) given the study design.

***Response:** We agree with the reviewer that the study design does not support causal or directional inference about transmission. We have revised the manuscript throughout to ensure that genomic analyses are framed as providing probabilistic evidence of shared reservoirs or exposure pathways, rather than direct or directional transmission.*

Specifically, we have:

- *Reframed genomics as complementary to, rather than replacing, conventional microbiological and epidemiological approaches (Introduction, Lines 95-97).*
- *Replaced causal language (e.g. “source of infection”, “direction of transmission”)*

with association-based terminology throughout.

- *Added a dedicated analysis framework section describing how genomic similarity and attribution models will be interpreted (lines 402-415).*
- *Added an explicit Limitations paragraph stating that the design does not support inference on transmission directionality (lines 538-548).*

These changes ensure that analytical claims are fully aligned with the study design.

DETAILED COMMENTS

Reviewer comment: Page 4 lines 99-101 The line "These studies, however, primarily relied on conventional culture or molecular diagnostics, and lacked the genomic resolution needed to investigate pathogen diversity, transmission pathways, and antimicrobial resistance (AMR) [50]" is scientifically incorrect in its assumption that only genomics can address these research questions.

Response: *We have revised this sentence to clarify that conventional culture-based and molecular diagnostics are effective for identifying pathogens, estimating burden, and characterising diversity at higher taxonomic levels, and that whole-genome sequencing provides complementary resolution at the within-species and strain level. The revised text no longer implies that genomics is uniquely capable of addressing these questions (Introduction, Lines 91-100).*

Reviewer comment: Page 4 lines 96-106: There are several large One Health studies on enteric pathogen diversity, sources, and transmission dynamics in Africa that predate this one and should
be referenced and discussed as far as how this study complements and differs.

Response: *We thank the reviewer for highlighting these important studies. We have expanded the Introduction and Discussion to explicitly reference and discuss the CAGED study (Ethiopia), PATHOME, and Urban Zoo (Kenya) (Introduction, Lines 104-113, Discussion, Lines 502-505).*

Reviewer comment: Page 6 lines 139 to 142: This section uses the words "the optimal study design" and then proceeds to name the three most common types of epidemiological designs, suggesting all epi design approaches are optimal designs. Please remove or make the point of this statement clearer.

Response: *This section has been rephrased to remove the implication that all epidemiological designs are optimal and to clarify the rationale for the chosen design.*

Reviewer comment: Page 6 lines 141-142 the primary subject identification procedure uses a case-control design with Medically attended diarrhea (MAD) as the criterion for case or control classification. Many studies, including the GEMS and MAL-ED studies cited in this manuscript and many others, have highlighted poor correlation between diarrhea and detection of pathogens in kids in high burden settings and high asymptomatic burden (mentioned on lines 84-85). Based upon those studies, enrolling a control based upon a lack of diarrhea symptoms gives the study team an approximately 25 to 40% chance of enrolling a control without some type of a subacute infection, depending upon subject age and geographic location. Many mild diarrhea cases or cases that do not meet WHO criteria (e.g. diarrhea twice in 24 hours) do not go to clinic and would be classified as a
health control in this study. Therefore, these lines should be revised to state the case control design provides valuable information about MAD risk factors, not infection risk factors.

Response: *We thank the reviewer for this important clarification. GETCampy is not designed to estimate epidemiological risk factors for infection or medically attended diarrhoea and does not undertake regression-based risk modelling.*

Instead, the case-control structure is used as a sampling framework to support comparative genomic analysis of symptomatic and asymptomatic Campylobacter carriage within shared households and communities. Inclusion of controls enables us to assess whether Campylobacter strains carried asymptomatically are:

- *genetically similar to those associated with medically attended diarrhoea,*
- *drawn from the same lineages or clonal complexes,*
- *and attributed to similar or distinct animal and environmental reservoirs.*

To avoid misunderstanding, we have revised the manuscript to:

- *Explicitly state that control samples are included to characterise asymptomatic carriage (Lines 190-192).*
- *Clarify that the study focuses on genomic contrast and population structure, not epidemiological risk estimation (Lines 178-185).*
- *Refine use of the term "risk" to refer to lineage-level pathogenic potential rather than host exposure.*

Reviewer comment: Page 6 lines 150 to 152: This suggests that there was a systematic sampling plan that would advise field staff on when and how to collect human feces, animal feces, or environmental samples. Standardized protocols are critical for ensuring enumerator beliefs and attitudes about sample collection do not bias the chances of detecting different types of bacteria, e.g., random hand rinses vs 5 minutes after handwashing. There are no details provided about how sampling was done.

Pages 7 to 11 Lines 170 to 340: The entire structure of this section does not flow clearly. Further, it does not present sufficient information that would let anyone repeat the study or understand what was done.
- There is no sample size section describing the intended number of subjects nor how that number was calculated.
- There is a paragraph 170-181 that is entitled “Participant selection and environmental sampling strategy” which refers to a case-control study, as in the introduction, but it provides no detail about the study design process. Health care centers are mentioned in the legend of Figure 2 so presumably recruitment and case enrollment happens there. Yet how enrollment was done and what eligibility and exclusion criteria were is not described which makes it problematic to understand who is actually considered a case. Cases are described as children with MAD, but health controls in the household are controls for Campylobacter positive MAD patients, implying there is a diagnosis process occurring between a MAD case presenting at clinic and confirmation that controls should be recruited. When and where is this Campy diagnosis happening? Does the case remain at the clinic until diagnosis is done (Fig 2 suggests enrollment is done at clinic) or does the case go home with household follow up being dailyed for some period of time until Campy diagnosis is complete? How long does the whole process take between diagnosis, case enrollment, control enrollment, and data collection?
- Did clinics enroll all cases, a proportion of cases, a number of cases per time interval, etcetera?
- Child age appears to be the only case eligibility criteria. Please confirm. No eligibility criteria are described for controls.
- The title “OBSERVATIONAL STUDY: genomic epidemiology and transmission of Campylobacter (GETCampy)” is inconsistently formatted relative to other sections. It also does not contain complete information and references a crosssectional follow-up, which

seems to simply be completion of the control enrollment/data collection part of the case-control study. The lack of a study timeline means that it is unclear when household visits and control enrolment happen relative to case identification nor are there details on how controls are selected and enrolled. Is the team enrolling everyone in the household as a control or picking one member as a representative control? What happens when all eligible controls can't be controls because they just had diarrhea? Would there be no control and thus the case child is disenrolled? How long are cases and controls enrolled in the study?
- Protocols for epidemiological studies are expected to report enough detail about the study design for public assessment of rigor, reproducibility, and transparency. Please reorganize and map paragraph content against the SPIRIT guidelines that were chosen as a reporting tool.

Response: *We have expanded and reorganised the Participant selection and environmental sampling strategy section (Lines 236-319) to include:*

- *Standardised sampling windows relative to clinic presentation,*
- *Clear definitions of human, animal, and environmental sampling units,*
- *Procedures for harmonised field training and SOP adherence,*
- *Explicit linkage to associated SOPs and data dictionaries.*

Reviewer comment: Page 10 Line 281-283 - This has nothing to do with the study approach. Please move to introduction or discussion or revise to make the approach detail apparent.

Response: *This text has been removed.*

Reviewer comment: The study design does not support inference about directionality of transmission.

Response: *As noted above, we have:*

- *Replaced causal language with probabilistic terminology throughout,*
- *Added a dedicated Analysis framework section (lines 402-415),*
- *Added an explicit Limitations paragraph addressing temporal ambiguity and bidirectional transmission (lines 538-548).*

Reviewer comment: Please create a distinct Analysis section that describes how each type of genomic evidence will be used to address objectives (diversity, pathogen sources, transmission pathways). Per the big picture comments above, either revise language throughout to remove suggestion of causal inference or clarify how bioinformatics approaches will use case-control(s) study design data to make conclusions about pathogen sources and pathways. With the described design, it is just as likely that the case shed a strain of Campylobacter (or other pathogens) in the household and infected the control(s), who could be asymptomatic but still shedding pathogens. The same question about directionality arises as far as whether the case is the victim or source of pathogens deteted in the environment and animals.

Response: *We have added a distinct Analysis framework (lines 402-415) section describing:*

- *Analysis of WGS and metagenomic data for diversity and population structure,*
- *Application of probabilistic and machine-learning attribution models,*
- *Integration of genomic and epidemiological metadata.*

Reviewer comment: Page 12 – The discussion should include a paragraph on limitations of the described study approach. Per comments about pre-existing CAGED/PATHOME/Urban Zoo pathogen transmission studies, how might the evidence from GetCampy be used alongside or address gaps in those studies to address the mutual goal of these studies to improve the use of effective disease control interventions?

Response: *We have added a dedicated Limitations paragraph explicitly addressing the constraints of the study design and discussing how GETCampy complements existing studies such as CAGED, PATHOME, and Urban Zoo by providing harmonised, multi-country genomic surveillance rather than directional transmission inference (lines 538-548).*

Reviewer comment: Page 106 - There is a data dictionary for a survey in the appendix, but no reference to when or how it is used in the recruitment, enrollment, and matriculation process.

Response: *We have clarified when and how the data dictionary is used during recruitment, enrolment, and data capture, and explicitly referenced it within the Design principles and methodologies section.*

Reviewer comment: A simple grammar review would be beneficial as there are some nonsensical statements, like "it is recommended that enteric bacterial pathogens should use a structured survey approach (line 137-138)" and "pathogens circulating in one health settings (lines 241-242). Bacteria cannot conduct surveys and all places are one health settings, so what is the counterfactual to this? Do the authors mean high disease burden settings?

Many of the pictures in the SOP are poor quality resolution and are not cited suggesting these are original pictures of bacteria plates (Fig 2) and stains and tests (Figs 4-8). All images replicated from standard microbiology reference guides (FDA BAM or EU EFSA or textbooks) should have credits as to source of the image and reuse approval.

Response: *We have conducted a full language and formatting review to correct unclear or nonsensical phrasing. In addition, all non-essential illustrative figures have been removed from the SOPs, with procedures now relying on text, tables, and QC criteria only, resolving concerns about image quality, provenance, and reuse permissions.*

Conclusion

We thank the reviewer for their thoughtful and detailed feedback. The revisions made in response have substantially improved the clarity, rigor, and transparency of the manuscript, and we believe the revised protocol now meets BMJ Open's expectations for reproducible observational study reporting.
